# Supplementary figures and images for: Breakthrough infections by SARS-CoV-2 variants boost cross-reactive hybrid immune responses in mRNA-vaccinated Golden Syrian hamsters
Source: PLoS Pathog. 2024 Jan 10;20(1):e1011805. doi: 10.1371/journal.ppat.1011805 (PMC10805310; doi:10.1371/journal.ppat.1011805)

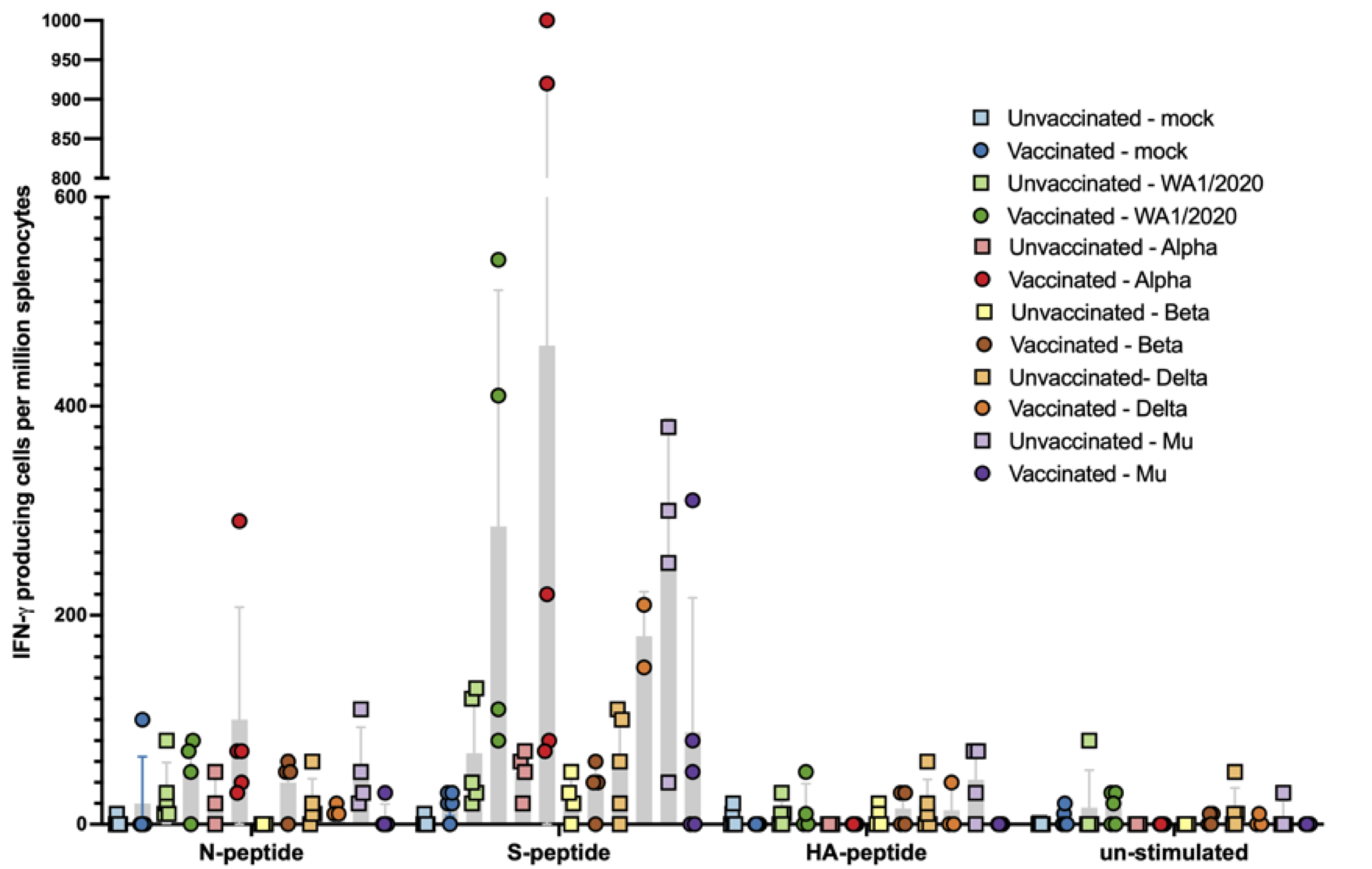

Supplement: S1 Fig — INF-y producing cells per million splenocytes after stimulation with (from left to right): N-peptide, S-peptide, HA-peptide and un-stimulated. (TIF) [file ppat.1011805.s001.tif]

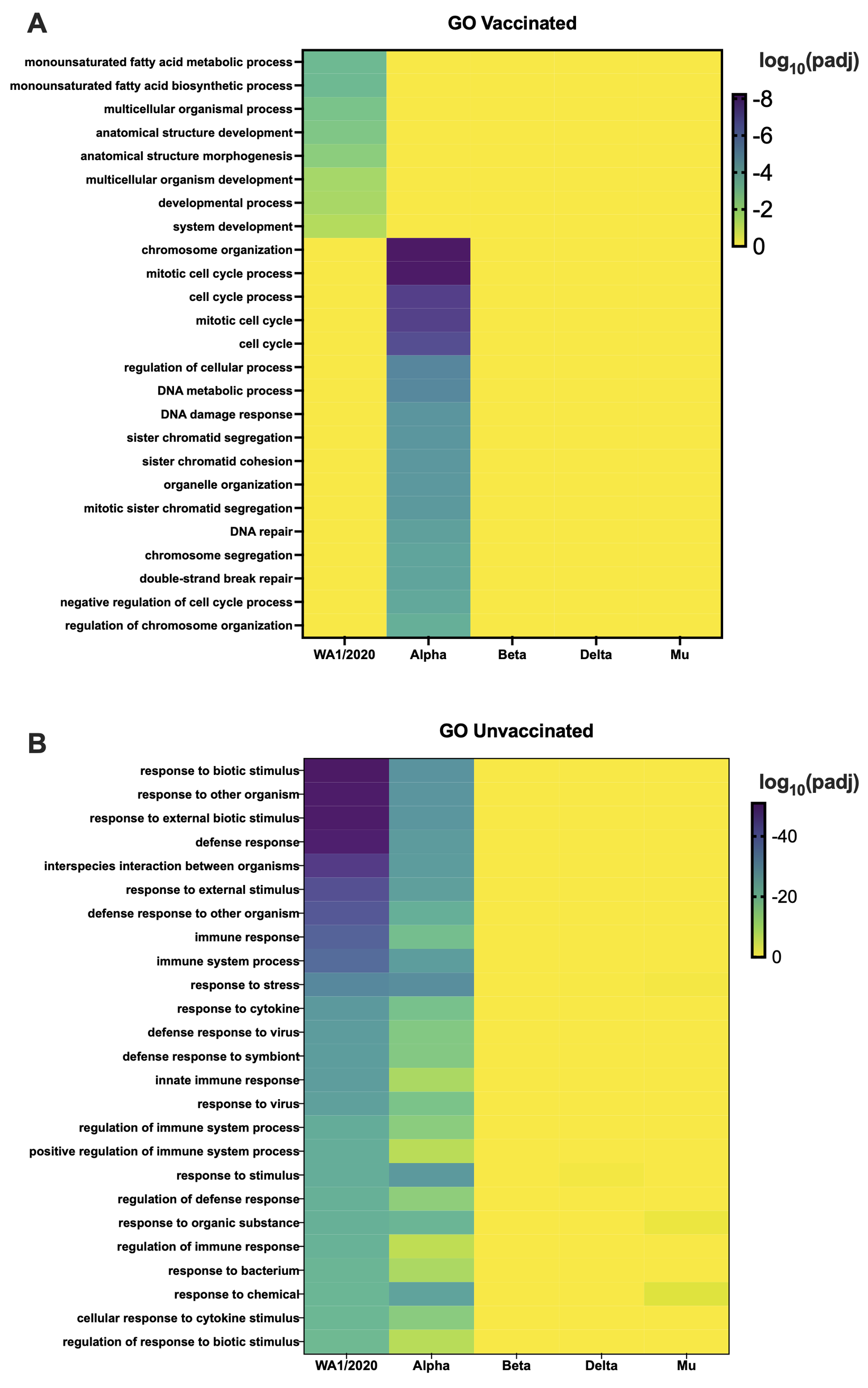

Supplement: S2 Fig — Top 25 most significantly upregulated biological processes based on significance in WA1/2020 challenged groups. (A) Upregulated biological processes in vaccinated animals compared to unvaccinated (B) Upregulated biological processes in unvaccinated animals compared to vaccinated. (TIF) [file ppat.1011805.s002.tif]

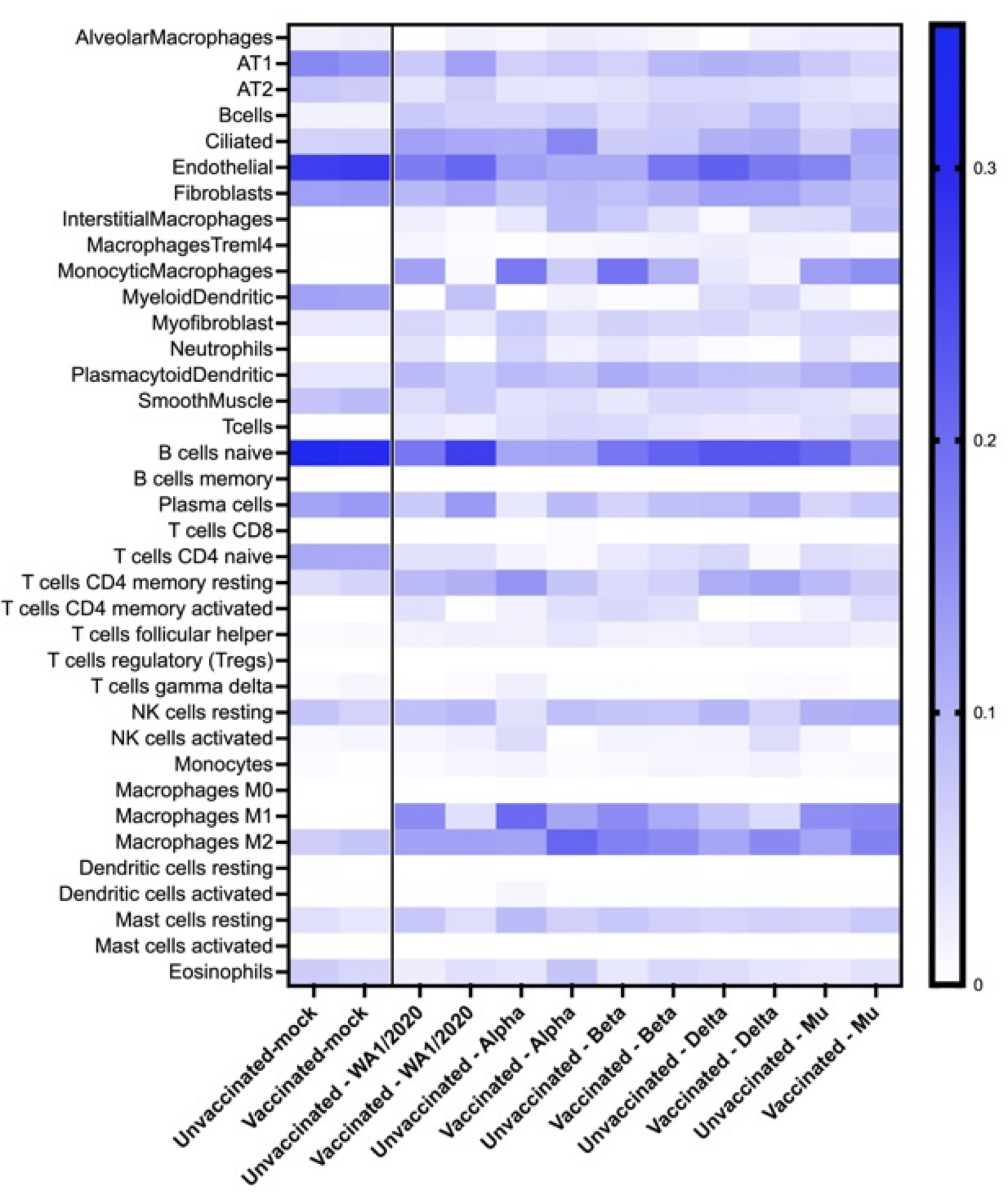

Supplement: S3 Fig — Scale represents estimated fraction of each cell type for each condition. (TIF) [file ppat.1011805.s003.tif]
